# Acute Pancreatitis as a Rare Complication of Plasmodium falciparum Malaria: A Case Report

**DOI:** 10.7759/cureus.116960

**Published:** 2026-09-26

**Authors:** Hussein Bagha, Tejal Patel, John Satish

**Affiliations:** 1 Department of Nephrology, Meghji Pethraj (MP) Shah Hospital, Nairobi, KEN; 2 Department of Internal Medicine, Meghji Pethraj (MP) Shah Hospital, Nairobi, KEN

**Keywords:** acute pancreatitis, plasmodium falciparum, red blood cell sequestration, severe malaria, systemic inflammatory response syndrome

## Abstract

While *Plasmodium falciparum* malaria is well-documented for its systemic complications, its association with acute pancreatic inflammation remains exceedingly rare and clinically elusive. The manifestation of acute pancreatitis during severe malaria infection presents a distinct diagnostic challenge, as overlapping constitutional symptoms can easily obscure pancreatic involvement. Understanding this unusual presentation is critical, as microvascular sequestration of parasitized erythrocytes and subsequent localized ischemia are thought to drive the underlying pancreatic injury, necessitating early clinical vigilance to prevent adverse outcomes.

This report details the clinical case of a patient diagnosed who was diagnosed with malaria. She was initially misdiagnosed with gastritis at a peripheral facility before malaria was identified and treated with intravenous artesunate. Persistent symptomatology of epigastric pain, nausea, and vomiting during the hospital stay led to a suspicion of acute pancreatitis, which was subsequently confirmed by laboratory tests and imaging. The patient improved and was successfully discharged.

This case highlights the importance of considering rare complications of malaria secondary to *P. falciparum* and *Plasmodium vivax *coinfection, particularly in endemic regions. A high index of suspicion in patients presenting with persistent abdominal symptoms would help to improve patient outcomes.

## Introduction

According to the World Health Organization (WHO)'s latest World Malaria Report, there were an estimated 263 million cases and 597,000 malaria deaths worldwide in 2023. This represents about 11 million more cases in 2023 than in 2022, with nearly the same number of deaths. Approximately 95% of the deaths occurred in the WHO African Region [1].

In Kenya, an average of 4.9 million outpatient cases of malaria were reported annually over the preceding five-year period, and annual incidence rose from 96 cases per 1,000 population to 105 cases per 1,000 between 2019 and 2023 [2]. Malaria prevalence varies across different epidemiological zones, ranging from 19% in lake-endemic areas to less than 1% in low-risk regions [2].

*Plasmodium falciparum* is one of the most widespread systemic protozoan pathogens, causing millions of clinical malaria cases annually [1-3]. It is the dominant malaria parasite in Kenya, with *Plasmodium vivax* prevalence about 2%, primarily in semi-arid regions [3,4]. *P. falciparum* is responsible for the most severe manifestations of the disease, including cerebral malaria, acute respiratory distress syndrome, acute kidney injury, myocarditis, hepatitis, hemolytic anemia, and other severe systemic complications [3-5]. On occasion, atypical complications may emerge, presenting a significant diagnostic and therapeutic challenge. Acute pancreatitis is one such manifestation of this illness, with only 12 cases reported as of 2016 [5]. The objective of this report is to describe the diagnostic approach and therapeutic interventions in a patient presenting with this rare complication with a background of *P. falciparum* and *P. vivax* coinfection.

This article was previously posted to the Research Square Preprint server on September 15, 2025 (doi.org/10.21203/rs.3.rs-7061844/v1).

## Case presentation

A 25-year-old female patient who was initially admitted to a peripheral facility presented to our facility with a one-week history of generalized malaise, paroxysmal fever, chills, global headache, and light-headedness. Additionally, she had developed nonradiating epigastric and periumbilical abdominal pain with associated nausea, vomiting, and one episode of large-volume loose stool, as well as dark-colored urine. Her medical history was notable for recurrent gastritis managed with over-the-counter self-medication. She had no recent travel to malaria-endemic areas and denied alcohol consumption or long-term prescription drug use. She was initially diagnosed with gastritis and was managed with proton pump inhibitors and antiemetics, which provided minimal relief. She was later diagnosed with malaria, seen on a peripheral blood film, at a peripheral facility, initiated on intravenous artesunate 2.4 mg/kg and referred to our facility for further management.

On examination, the patient was found to have confusion, dehydration, hypotension with a blood pressure of 96/54 mmHg, and a tachycardia of 112 beats/minute. She had moderate pallor and mild jaundice. She had marked epigastric tenderness and reduced bilateral bibasal breath sounds.

Her lipase level was noted to be three times the upper limit of normal, and a diagnosis of acute pancreatitis was made. Her abdominal ultrasound showed gallbladder sludge with no cholelithiasis. She had an aspartate aminotransferase of 262.6 U/L, an alanine aminotransferase of 75.3 U/L, a gamma-glutamyl transferase of 45.1 U/L, an alkaline phosphatase of 91.6 U/L, and a direct bilirubin of 32.1 umol/L. She had a normal head computed tomography (CT) scan. Her serum urea was elevated. Her peripheral blood slide (thick and thin malaria slide) showed a 1% parasitemia with *P. falciparum* trophozoites. Her peripheral blood film revealed normocytic, normochromic anemia, neutrophils exhibiting toxic granulation, lymphopenia, and a moderate reduction in the platelet count. The parameters immediately relevant to her status are tabulated in Table 1.

**Table 1:** Relevant laboratory and radiological investigation results from day of admission CT: computed tomography

Parameter	Day 1	Day 3	Day 10	Reference ranges
Calcium (mmol/L)	1.89	1.95	-	2.02-2.65
Urea (mmol/L)	27.78	16.25	7.69	1.7-8.3
Creatinine (µmol/L)	173.3	144.6	75.1	35-120
Lipase (U/L)	528.9	282.6	-	0-190
Amylase (U/L)	267.7	235.6	-	0-100
Triglycerides (mmol/L)	6.05	-	-	<2.3
Malaria parasites	1% parasitemia	No parasites	No parasites	No parasites
Abdominal ultrasound (15/06)	Biliary sludge	-	-	Normal
CT scan abdominopelvic (17/06)	Acute pancreatitis, moderate ascites, atypical pneumonia, bilateral pleural effusion	-	-	Normal

She was initiated on intravenous artesunate (2.4 mg/kg) and supportive medications. Ten hours after admission, her care was escalated to the high dependency unit due to persistent hypotension. A gastroenterology consult was additionally sought. The plan of care remained to optimize her supportive care.

Noncontrast abdominal and pelvic CT imaging showed a diffusely enlarged pancreas (Figure 1), with parenchymal necrosis not excluded, as it was a noncontrast CT scan, with contrast enhancement being deferred in view of her renal dysfunction. There was moderate inflammatory thickening of the peripancreatic fat. Fluid collections were noted around the pancreas as well as in the abdomen and pelvis. Bilateral pleural effusions were also noted (Figure 2).

**Figure 1:**
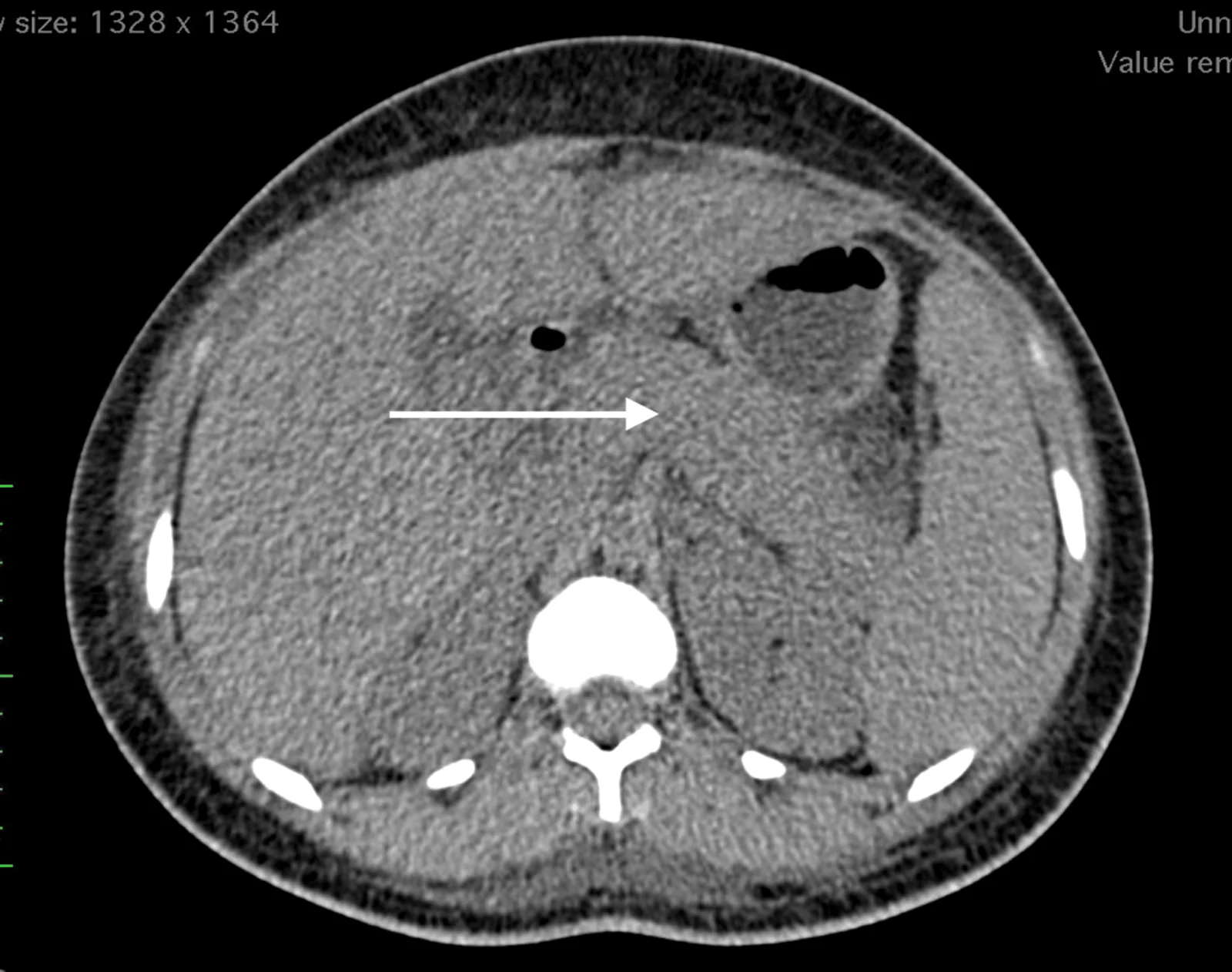
CT scan abdomen showing diffuse enlargement of the pancreas with moderate inflammatory thickening of peripancreatic fat planes (white arrow) CT: computed tomography

**Figure 2:**
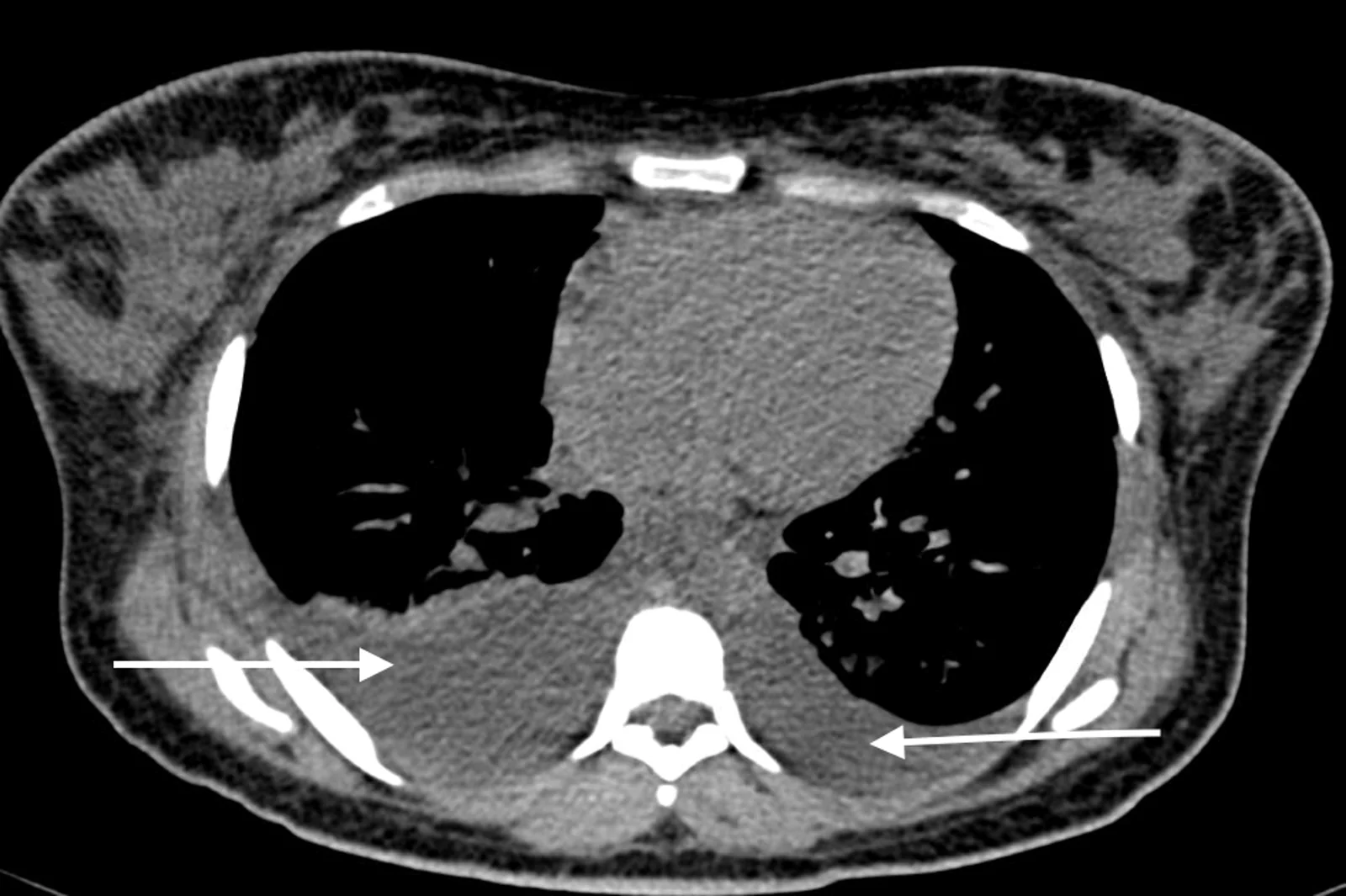
CT scan showing bilateral pleural effusions with anteroposterior depths of 4 cm (right) and 3 cm (left) as shown by the white arrows CT: computed tomography

Her autoimmune panel (extractable nuclear antigen profile/antinuclear antibody/anti-double-stranded deoxyribonucleic acid) was negative, and she was found to have hypertriglyceridemia (6.05 mmol/L), albeit this level was not high enough to warrant overt caution. An arterial blood gas done at 48 hours after admission showed a base deficit of 4.2 mmol/L and a partial pressure of arterial oxygen of 11.3 kPa.

Her Bedside Index for Severity of Acute Pancreatitis (BISAP) score was calculated within the first 24 hours. She scored 4 points based on the presence of bilateral pleural effusions, impaired mental status, systemic inflammatory response syndrome (SIRS; tachycardia, fever and tachypnea), and elevated blood urea nitrogen (77.8 mg/dL). This placed her in the high-risk category, warranting intensive monitoring and early intervention.

She received supportive management for acute pancreatitis, including intravenous fluids, antiemetics, proton pump inhibitors, and transfusions for her dropping hemoglobin levels when required. Her subsequent malaria slides were negative, and she was stepped down to the ward setting on day 6 of admission.

Her tropical fever screen (polymerase chain reaction (PCR) test) was positive for *P. vivax*, and she was initiated on primaquine at 0.5 mg/kg for 14 days. With supportive care consisting of fluid resuscitation, analgesia, and nutrition on demand, her condition improved, and she was discharged on day 10 (Table 2).

**Table 2:** Timeline of clinical events CT: computed tomography; HDU: high dependency unit

Day	Fever	Parasitemia	Artesunate	Artemether-lumefantrine	Primaquine	Epigastric pain	Lipase (U/L)	Amylase (U/L)	Notes
0	37.7	-	1st dose	-	-	Present (1 week)	-	-	Peripheral facility
1	36.4	1%	2 doses	-	-	Present	528.9	267.7	Admission date
2	36.7	-	1 dose	-	-	Present	-	-	Transferred to HDU
3	36.7	No parasites	1 dose	-	-	Present	282.6	235.6	CT imaging performed
4	36.8	-	1 dose	-	-	Improving	-	-	-
5	-	-	-	1 dose	1 dose	Improving	-	-	Polymerase chain reaction result positive for P. vivax; artemether-lumefantrine started
6	-	-	-	2 doses	1 dose	Improving	-	-	Ward step-down
7	-	-	-	2 doses	1 dose	Improving	-	-	Clinical recovery
8	-	No parasites	-	1 dose	1 dose	Resolved	-	-	End of artemether-lumefantrine
9	37.0	-	-	-	1 dose	Resolved	-	-	None
10	-	-	-	-	1 dose	Resolved	-	-	Discharged

## Discussion

Acute pancreatitis has a high morbidity rate, with significant hospital admissions. A study published in the American Journal of Gastroenterology reported that the incidence of acute pancreatitis in the United States is estimated to be around 34 cases per 100,000 persons per year [6]. The estimated global mortality rate is about 1.60 per 100,000 person‑years [7]. Complications include organ failure, sepsis, and necrotizing pancreatitis, which increase the severity of the disease and length of hospital stay [8].

Malaria remains a devastating global health problem [9]. Ironically, the overall incidence of pancreatitis in malaria is extremely rare, with reported cases constituting only about 1.5% of all malaria-related complications [10]. Malaria-induced pancreatitis is typically severe, often associated with other complications such as renal failure, respiratory distress, and cerebral malaria [11]. Mortality rates for malaria-induced pancreatitis vary widely, but in the most severe cases of falciparum malaria with pancreatitis, the mortality rate can approach 30%-40% [11]. A study by Barman et al. reported that, in malaria-induced pancreatitis, the mortality rate was around 20%-30%, especially if there were delays in diagnosis or if the condition was complicated by multiorgan failure [12]. This rare manifestation of malaria arises from multifactorial etiologies as elucidated below.

One major contributing mechanism is sequestration of *P. falciparum*-infected red blood cells within pancreatic capillaries. These cells express a protein called *P. falciparum* erythrocyte membrane protein 1, which facilitates their adherence to the endothelial lining of blood vessels. They attach to several endothelial receptors, including cluster of differentiation 36, intercellular adhesion molecule 1, vascular cell adhesion molecule 1, thrombospondin, and histidine-rich protein. The result is mechanical blockage of the capillaries and a phenomenon known as rosetting, whereby infected and uninfected red blood cells clump together, further obstructing blood flow and leading to intraparenchymal hypoxia and ischemia [12].

When blood flow is restored after such ischemic episodes, reperfusion injury may occur. This process involves generating reactive oxygen species, which induce oxidative stress and can trigger premature activation of digestive enzymes within pancreatic acinar cells, leading to autodigestion and inflammation [13].

In addition to local circulatory disturbances, severe *P. falciparum* malaria is characterized by hemolysis. This releases free hemoglobin into the circulation, which further damages the vascular endothelium and impairs perfusion. At the same time, the body mounts a strong inflammatory response, producing cytokines such as tumor necrosis factor-alpha, interleukin (IL)-1, IL-6, and IL-8. These cytokines further stimulate endothelial cells, increase adherence of infected red blood cells to the vessel wall, and aggravate the microvascular obstruction [10-12].

The infection can also trigger a systemic inflammatory reaction similar to that observed in sepsis. This increases blood vessel permeability and allows immune cells to infiltrate pancreatic tissue, amplifying local inflammation and damage [12].

Histopathological examinations from autopsies have provided direct evidence of pancreatic involvement. These studies reveal interstitial edema, acinar necrosis, inflammatory cell infiltration, hemorrhages, and visible accumulations of parasitized red blood cells within pancreatic capillaries [10].

In addition, a BISAP score of 3 or more is associated with increased risk of complications and mortality [14]. The SIRS component of the BISAP score is defined by the presence of at least two of the following: temperature >38°C or <36°C, heart rate >90/minute, respiratory rate >20/minute or partial pressure of arterial carbon dioxide of <32 mmHg, and abnormal leukocyte count (>12 × 10⁹/L, <4 × 10⁹/L, or >10% bands). Our patient met three criteria (fever, tachycardia, and tachypnea), fulfilling SIRS and contributing 1 point to her BISAP score.

The proposed mechanisms of malaria-induced pancreatitis are reflected by the patient's clinical course. Microvascular obstruction by parasitized red blood cells likely contributed to pancreatic ischemia, supported by CT findings of an enlarged, inflamed pancreas and elevated pancreatic enzymes. Reperfusion injury and cytokine-mediated inflammation leading to autodigestion of the pancreas explain her severe abdominal pain, pleural effusions, elevated C-reactive protein, and procalcitonin. Hemolysis and thrombocytopenia were evident from her anemia and blood film, while renal dysfunction worsened her BISAP score and limited contrast-enhanced imaging [14]. Additionally, hypertriglyceridemia may have acted as a secondary insult contributing to the severity of pancreatitis; however, the literature states that the risk of this happening is exponentially increased with triglyceride levels that exceed 11.3 mmol/L [15]. Apart from that, SIRS likely contributed to the patient’s persistent hypotension. Collectively, these findings illustrate how the interplay of the pathophysiological processes involved culminated in acute pancreatitis in her case.

The diagnosis of acute pancreatitis in our patient was established in accordance with the Revised Atlanta Classification [16]. Our patient fulfilled all three criteria, presenting with severe epigastric pain, markedly elevated serum lipase (528.9 U/L; >3× upper limit of normal), and imaging findings consistent with acute pancreatitis on noncontrast-enhanced CT. Although the diagnosis was already supported by clinical and biochemical parameters, CT was pursued to evaluate the extent of disease, assess for local complications, and clarify the etiology. In terms of severity, the Revised Atlanta Classification defines moderately severe disease as transient organ failure (<48 hours) and/or the presence of local complications. Our patient demonstrated transient renal impairment (creatinine >170 µmol/L resolving within 48 hours) and ascites on CT, fulfilling the criteria for moderately severe acute pancreatitis. We considered viral causes of pancreatitis less likely given the absence of prodromal viral illness, systemic viral features, relevant exposure history, or immunosuppression.

Although drug-induced pancreatitis is a recognized entity, our patient had no recent exposure to high-risk agents such as azathioprine, valproate, sulfonamides, or thiazides. Moreover, there was no temporal relationship between medication use and symptom onset, and the clinical course was not influenced by drug continuation or withdrawal. Therefore, a drug-related etiology was considered unlikely.

Although glucose-6-phosphate dehydrogenase deficiency (G6PD) is distributed throughout Kenya, the most common variant is the African A- type, which generally confers mild-to-moderate enzyme deficiency, with severe variants, such as those seen in the Mediterranean and parts of Asia, being rare in our setting [17]. In our patient, there was no clinical history suggestive of hemolysis and no prior adverse drug reactions, which reduced the likelihood of a severe G6PD variant. While testing was not performed prior to primaquine administration, the regional epidemiology and clinical context suggest a relatively low probability of significant hemolysis [17]. Additionally, our patient required urgent initiation of treatment due to her clinical status and required us to make a risk vs. benefit assessment as per international guidelines [17]. Her hemoglobin was stable at the time and monitored daily. In addition, she was financially constrained, and the G6PD test results would have taken a week to return, a delay we could not afford given her clinical status.

Her antimalarial regimen consisted of intravenous artesunate dosed at 2.4 mg/kg, given at 0, 12, and 24 hours, followed by once daily until she could tolerate oral therapy (day 5), at which point she was switched to artemether-lumefantrine dosed at 80 and 480 mg, respectively, given twice daily over three days. Her primaquine was dosed at 0.5 mg/kg once daily for a total of 14 days [2].

One would be pressed to look into the possibility of biliary sludge/microlithiasis contributing to the patient’s pathology. Although the patient had conjugated hyperbilirubinemia and elevated cholestatic enzymes, ultrasound showed no dilatation of the central biliary ducts, common hepatic duct, or common bile duct; a well-distended gallbladder with uniform mural thickness; or no pericholecystic fluid. However, low-level sludge was noted. Additionally, the patient had a single episode of transaminitis without recurrence and strong alternative risk factors. Taken together, these findings make microlithiasis an unlikely etiology in this case.

Per international guidelines, the absence of imaging evidence of ductal obstruction excludes a diagnosis of obstructive jaundice [18]. As such, there was no magnetic resonance cholangiopancreaticography done during her follow-ups in the clinic as her blood work and her symptomatology had improved. The cost implication for the investigation was not justifiable, especially following her abdominopelvic ultrasound findings.

The patient was found to have mixed Plasmodium infection.* P. falciparum* was identified on peripheral blood smear microscopy, which remains the gold standard in malaria diagnosis due to its ability to quantify parasitemia and distinguish between species, although its sensitivity is operator-dependent. A malaria antigen test was additionally used and had a positive result. *P. vivax* was detected using a tropical fever PCR panel, which offers superior sensitivity (over 95%) and specificity (>98%) compared with microscopy, particularly in cases of low parasitemia or mixed infections where non-falciparum species are often underrecognized [2]. The coexistence of *P. falciparum* and *P. vivax* has important clinical implications: while *P. falciparum* is associated with severe disease and complications, *P. vivax* can contribute to morbidity through recurrent relapses due to dormant liver hypnozoites. This dual infection likely influenced both illness severity and the therapeutic approach, as radical cure with primaquine was required to target *P. vivax* hypnozoites while ensuring adequate treatment for *P. falciparum* [2].

Clinicians in malaria-endemic regions should maintain a high index of suspicion for signs of acute pancreatitis in patients with *P. falciparum* infection who develop upper abdominal pain, nausea, vomiting, or elevated serum levels of pancreatic enzymes such as amylase and lipase. Acute pancreatitis should be considered even when common causes have been excluded [19,20].

## Conclusions

Despite being a preventable and treatable disease, malaria contributes to significant morbidity and mortality in Kenya. While complications such as cerebral malaria and acute kidney injury are well known, acute pancreatitis remains an underreported and likely under-diagnosed consequence of malaria. This case highlights its multifactorial pathophysiology as well as the importance of maintaining a broad differential in malaria patients presenting with abdominal symptoms. Vigilance of and prompt investigation is likely to lead to improved outcomes in what may otherwise be a life-threatening complication.
